# Outcomes from cardiac surgery in Jehovah’s witness patients: experience over twenty-one years

**DOI:** 10.1186/s13019-016-0455-6

**Published:** 2016-04-14

**Authors:** Sotirios Marinakis, Philippe Van der Linden, Redente Tortora, Jacques Massaut, Charalampos Pierrakos, Pierre Wauthy

**Affiliations:** Department of Cardiac Surgery, Brugmann University Hospital, Laeken, Belgium; Department of Anesthesiology, Brugmann University Hospital, Laeken, Belgium; Department of Intensive Care Unit, Brugmann University Hospital, Laeken, Belgium; Service de Chirurgie cardiaque, CHU Brugmann Université Libre de Bruxelles, 4 place A van Gehuchten, 1020 Laeken, Belgium

**Keywords:** Bleeding, Blood transfusion, Cardiopulmonary bypass, Cell saver, Reoperation

## Abstract

**Background:**

Cardiac surgery in Jehovah’s Witnesses may be challenging during the operation and postoperative period given their refusal of blood products. The aim of this study was to document our center’s experience with Jehovah’s Witnesses undergoing major cardiac surgery and to compare surgical outcomes with a matched control group.

**Methods:**

We retrospectively reviewed the demographic, perioperative, and in-hospital postoperative data for 31 Jehovah’s Witness patients undergoing surgery from 1991 to 2012 and compared findings with a control group of 62 patients of the same sex and age, who underwent the same type of operations in the same period. Early mortality, major in-hospital morbidity, laboratory findings, and hospital stays were compared between groups.

**Results:**

Demographic data were similar between groups, except that more patients in the Jehovah’s Witness group had extracardiac arteriopathy compared with controls (*p* = 0.04). There was no difference in predicted mortality, calculated by the Euroscore II, between groups (2.8 ± 3.3 in study group versus 2.4 ± 2.2 in control group, *p* = 0.469). For postoperative outcomes, there were no differences between Jehovah’s Witnesses versus controls in hospital mortality (3 % versus 2 %, *p* = 0.548), total drain loss (847 ± 583 mL versus 812 ± 365 mL, *p* = 0.721), mechanical ventilation time (1.26 ± 2.24 versus 0.89 ± 0.55 days, *p* = 0.218), intensive care unit stay (4.3 ± 3.9 versus 3 ± 1.4 days, *p* = 0.080), and hospital stay (12.9 ± 7.6 versus 10.9 ± 6.6 days, *p* = 0.223).

**Conclusions:**

Outcomes after cardiac surgery are similar between Jehovah’s Witnesses and general population, in centers applying rigorous blood patient management protocols.

## Background

Jehovah’s Witnesses refuse the transfusion of blood products, based on Biblical passages citing to “abstain from food polluted by idols, from sexual immorality, from the meat of strangled animals and from blood” (Acts 15:19–20). Before major surgery, these patients are confronted with the stress of both the operation and the potential need for transfusion. The role of the health care provider is to assure the physical and psychological welfare of patients while respecting their philosophical and religious beliefs. However, despite the important evolution of blood preservation techniques, there is still a great risk for transfusion in cardiac surgery. Transfusion rates during hospitalization for a cardiac surgery procedure vary from 25 to 95 % [1–3]. In this context, cardiac surgery in Jehovah’s Witnesses represents a real challenge, necessitating a close collaboration between the surgical, anesthesiological, and medical teams. However, due to the Jehovah’s Witnesses’ “natural experiment of blood abstention”, the overall need for transfusion during cardiac surgery should be reevaluated [4]. Recent studies did not find any difference in terms of mortality between Jehovah’s Witnesses and a control group after cardiac surgery [4–7]. Moreover, the deleterious effects of blood transfusions in terms of postoperative morbidity and long term mortality after cardiac surgery, along with the cost and shortage of available blood products, suggest tighter transfusion protocols [1, 2, 8–11]. In this context, the investigation of surgical outcomes in Jehovah’s Witnesses helps to evaluate the limits of transfusion safety and cut off points for transfusion, while improving the hospital care of this challenging group of patients [12]. The aim of this study was to document our center’s experience with complex cardiac surgery, including reoperations and urgent operations, in a non-selected Jehovah’s Witness group and to compare surgical outcomes with a matched control group.

## Methods

The current retrospective study was carried out by the Cardiac Surgery Department of Brugmann University Hospital in Brussels. All adult Jehovah’s Witness patients operated in our hospital from 1991 to 2012 (*n* = 31) were identified through our computerized database. Baseline demographics, perioperative data, postoperative outcomes, and laboratory data (Tables 1, 2, 3 and 4) were recorded after consultation of each patient’s chart and admission medical history. For each of the 31 Jehovah’s Witnesses, the previous and the next patient of the same sex who underwent the same surgical procedure were included in the control group if their age difference from the Jehovah’s Witness patient was ≤ 5 years. All patients were operated on by the same surgical team consisting of four senior surgeons (Table 1).

**Table 1:** Patients’ demographics

	Jehovah’s Witnesses (*n* = 31)	Control Group (*n* = 62)	*p* Value
Age in years	62 ± 15	62 ± 14	0.873
BMI (kg/m^2^)	27.4 ± 4.3	26.2 ± 4.0	0.182
Women	10 (32 %)	20 (32 %)	1.000
Number of operations per surgeon (1, 2, 3,4)	13, 10, 4, 4	15, 27, 12, 8	0.344
Euroscore II	2.80 ± 3.34	2.38 ± 2.20	0.469
Preoperative Cockcroft’s CC in mL/min	84 ± 29	82 ± 27	0.776
Renal insufficiency (Cockcroft’s CC <85 mL/min)	16 (52 %)	34 (55 %)	0.769
Diabetes	8 (26 %)	15 (24 %)	0.865
Recent smoker	3 (10 %)	17 (27 %)	0.062
Hypertension	18 (58 %)	30 (48 %)	0.379
Hypercholesterolemia	19 (61 %)	36 (58 %)	0.765
Positive family history	10 (32 %)	18 (29 %)	0.749
Cerebrovascular accident	4 (13 %)	5 (9 %)	0.457
Peripheral vascular disease	8 (26 %)	6 (10 %)	0.040
Previous myocardial infarction	6 (19 %)	11 (18 %)	0.850
Recent myocardial infarction (<90 days)	3 (10 %)	6 (10 %)	1.000
COPD	6 (19 %)	6 (10 %)	0.189
NYHA			0.452
I	6 (19 %)	7 (11 %)	-
II	12 (39 %)	21 (34 %)	-
III	10 (32 %)	30 (48 %)	-
IV	3 (10 %)	4 (6 %)	-
Preoperative LVEF (%)	62 ± 11	59 ± 14	0.340
Preoperative AF	5 (16 %)	9 (15 %)	0.838
Reoperation	3 (10 %)	8 (13 %)	0.650
Urgent status	7 (23 %)	6 (10 %)	0.091

*BMI* body mass index, *CC* creatinine clearance, *Recent smoker* current smoker or ex-smoker for less than 5 years, *COPD* chronic obstructive pulmonary disease, *NYHA* New York heart association score, *AF* atrial fibrillation, *LVEF* left ventricular ejection fraction

**Table 2:** Type of surgery

Type of operation	Jehovah’s Witnesses (*n* = 31)	Control group (*n* = 62)
CABG	15 (48 %)	30 (48 %)
CABG beating heart	2 (6 %)	4 (6 %)
AVR	5 (16 %)	10 (16 %)
MVR	2 (6 %)	4 (6 %)
Double VR	2 (6 %)	4 (6 %)
Combined VR + CABG	3 (10 %)	6 (10 %)
Bentall	1 (3 %)	2 (3 %)
AAR	1 (3 %)	2 (3 %)

*CABG* coronary artery bypass graft, *AVR* aortic valve replacement, *MVR* mitral valve replacement, *VR* valve replacement, *AAR* ascending aortic replacement

**Table 3:** Laboratory findings

	Hb (g/dL)			Plat (× 1000/mm^3^)			Cr (mg/dL)		
	JhW	CG	*p*	JhW	CG	*P*	JhW	CG	*p*
D_−1_	14.2 ± 1.6	13.7 ± 1.7	0.174	226.4 ± 55.2	233.7 ± 75	0.640	1.13 ± 0.29	1.08 ± 0.20	0.272
D_0_	10.6 ± 1.8	10.4 ± 1.3	0.478	156.3 ± 38.9	156.0 ± 47.4	0.974	1.13 ± 0.29	1.05 ± 0.22	0.112
D_1_	11.0 ± 2.0	10.9 ± 1.5	0.849	179.7 ± 50.8	179.3 ± 55.4	0.977	1.28 ± 0.48	1.15 ± 0.30	0.123
D_2_	10.5 ± 2.2	10.5 ± 1.2	0.995	177.9 ± 66.3	167.6 ± 58.3	0.468	1.18 ± 0.55	1.03 ± 0.41	0.160
D_5_	10.7 ± 2.5	11.0 ± 1.7	0.513	220.1 ± 85.5	227.5 ± 87.7	0.732	1.12 ± 0.53	1.03 ± 0.50	0.445
D_7_	10.7 ± 2.5	11.4 ± 1.8	0.218	298.9 ± 93.1	329 ± 106.7	0.301	1.17 ± 0.67	1.13 ± 0.69	0.862

*JHW* Jehovah’s witnesses, *CG* control group, *Hb* hemoglobin, *Plat* platelets, *Cr* creatinine, *D*_−1_ preoperative, *D*_0_-*D*_7_ Day 0-Day 7

**Table 4:** Postoperative outcomes

	Jehovah’s Witnesses	Control group	*P* value
Time of ECC in min	104 ± 35	98 ± 36	0.432
Cross Clamp Time in min	61 ± 27	59 ± 29	0.721
Perioperative blood loss in mL	209 ± 235	308 ± 367	0.178
Drain 24 h in mL	644 ± 458	582 ± 215	0.481
Drain total in mL	847 ± 583	812 ± 365	0.721
Hours post drain ablation	46.3 ± 9.8	45.7 ± 12.1	0.811
LVEF postop (%)	57 ± 8	58 ± 10	0.912
Mechanical ventilation in days	1.26 ± 2.24	0.89 ± 0.55	0.218
ICU stay in days	4.3 ± 3.9	3.0 ? ± 1.4	0.080
Postoperative length of stay in days	12.9 ± 7.6	10.9 ± 6.6	0.223
IABP	1 (3 %)	0	0.333
Acute MI	0	1 (2 %)	1.000
Stroke	0	0	-
New onset AF	11 (35 %)	11 (18 %)	0.058
New AF on discharge	0	3 (5 %)	0.548
Max Cr postop (mg/dL)	1.43 ± 0.60	1.32 ± 0.60	0.378
Min Cockcroft’s CC postop (mL/min)	63.8 ± 32.3	64.8 ± 24.8	0.865
Hemodialysis	1 (3 %)	0	0.333
Hemorrhage related re-exploration	2 (6 %)	1 (2 %)	0.257
Mediastinitis	1 (3 %)	1 (2 %)	1.000
Serious infectious complications	5 (16 %)	3 (5 %)	0.112
Transfused patients	0	17 (27.4 %)	0.001
Units of RBC	0	39	-
Units of RBC pp	0 ± 0	0.63 ± 1.5	0.002
Operative mortality	1 (3 %)	1 (2 %)	0.548

*ECC* extracorporeal circulation, *ICU* intensive care unit, *LVEF* left ventricular ejection fraction, postop = postoperative, *RBC* red blood cells, *IABP* intra-aortic balloon pump, *MI* myocardial infarction, *Max* maximum, *Cr* creatinine, *Min* minimum, *CC* creatinine clearance, *pp* per patient, *Serious infectious complications* Pneumonia, Mediastinitis, Bacteremia

The same anesthesia protocol, surgical techniques, extracorporeal circulation, myocardial protection, and blood salvage techniques were used for both groups. A coated circuit with open venous reservoir and a centrifugal pump was used for extracorporeal circulation. Priming, consisted of only 1 L of colloid solution (modified fluid gelatin) and 150 mg of 20 % mannitol. Whenever patient’s hemodynamic state permitted it, retropriming technique was used to further reduce hemodilution. All the patients were maintained with an hematocrit up to 24 % and hemofiltration was systematically performed. All the procedures were achieved under mild hypothermia (between 32 and 33 °C). The cardiopulmonary flow rate was maintained between 2,8 and 3 L/min/m2 and the FiO2 (inspired oxygen fraction) was adapted to maintain a SvO2 (venous saturation in oxygen) above the 75 % threshold.

From 1991 to 2007, aprotinin was routinely administered in the priming solution as an antifibrinolytic agent and replaced thereafter by tranexamic acid [13]. For all operations where the ascending aorta was opened, retrograde cardioplegia was administered. For operations with an intact aortic root and competent aortic valve, anterograde cardioplegia was administered from the ascending aorta. From 1991 to 2010, a modified Saint Thomas crystalloid cold cardioplegia was used, subsequently replaced by a cold blood cardioplegia (8 °C) diluted with 23 % crystalloid solutions. Significant blood loss was systematically processed with a Cell Saver. For the Jehovah’s Witness group, a continuous connection between the Cell Saver’s washed blood bag and the patient’s peripheral intra-venous infusion line was used in accordance with their beliefs for continuous blood flow.

After 2003, all Jehovah’s Witness patients undergoing elective surgery were evaluated preoperatively to optimize their red blood cell volume. When hemoglobin was less than 13 g/dL, a total of three subcutaneous injections of 40 000 units of erythropoietin and intravenous iron were administered 3, 2, and 1 week before the operation. Control group patients did not receive any erythropoietin treatment, as it is not reimbursed by the Belgium national health care system for cardiac surgery. Oral anticoagulation therapy was routinely discontinued 3 days before surgery and replaced with fractionated heparin. Platelet anti-aggregation therapy was routinely discontinued 7 days before elective surgery cases [9, 10].

Univariate analysis of demographics as well as preoperative, operative, and postoperative in-hospital variables was performed between the two groups. Comparisons between continuous variables were performed using the Student *t*-test after the Levene’s test for equality of variances. Dichotomous variables were compared using the χ^2^ or the Fisher’s exact test for cell counts < 5. A *p* value ≤ 0.05 was considered significant and all tests were 2-sided. All parametric data are presented as the mean value ± standard deviation. Statistical analysis was performed with SPSS 20.0.

## Results

In our study, the Jehovah’s Witness group underwent a variety of cardiac surgery procedures, included reoperations and urgent procedures. The control group was selected based on the type of surgical procedure as described in the methods (Table 2). Demographic characteristics of Jehovah’s Witnesses versus the controls are presented in Table 1. Univariate analysis showed that the groups had similar demographics and preoperative characteristics. The Euroscore II was calculated for both groups and there was no difference in predicted mortality (*p* = 0.469). The left ventricular ejection fraction was also similar between groups (*p* = 0.340). The only significant difference between groups was that Jehovah’s Witnesses had more peripheral vascular disease than controls (*p* = 0.040, Table 1).

Hemoglobin, platelet, and serum creatinine values were compared between groups one day preoperatively and on days 0, 1, 2, 5, and 7 postoperatively and there was no significant difference at any time between groups (Table 3). Perioperative variables and postoperative outcomes are grouped in Table 4. No difference was observed in the time of extracorporeal circulation, aortic clamping, or perioperative blood loss. Drains were withdrawn within two days after the operation and their output was similar between groups (*p* = 0.721). One patient from each group underwent a reoperation for mediastinitis (*p* = 1.000). Two reoperations for hemorrhage or tamponade were performed in Jehovah’s Witnesses versus one in the control group (*p* = 0.257). No significant difference was observed in postoperative renal function, as defined by the maximum postoperative serum creatinine (*p* = 0.378) and the minimum postoperative creatinine clearance (*p* = 0.865). One Jehovah’s Witness developed severe renal failure at day1 necessitating hemodialysis. This patient developed multi-organ failure in sepsis post-mediastinitis and died in the intensive care unit on day 37 after a triple coronary artery bypass. This patient suffered from insulin depending diabetes with a preoperative logistic euroscore II of 15.09. Preoperative creatinine level and estimated creatinine clearance were respectively 2,04 mg/dL and 30,0 mL/min. He was operated in an urgent status because of a non ST elevation myocardial infarction. Minimal hemoglobin level during extracorporeal circulation was 9,4 gr/dL and minimal hemoglobin level the day of the procedure was 8,4 gr/dL. The hemoglobin level rose progressively up to 12,4 gr/dL at postoperative day seven.

**Fig. 1:**
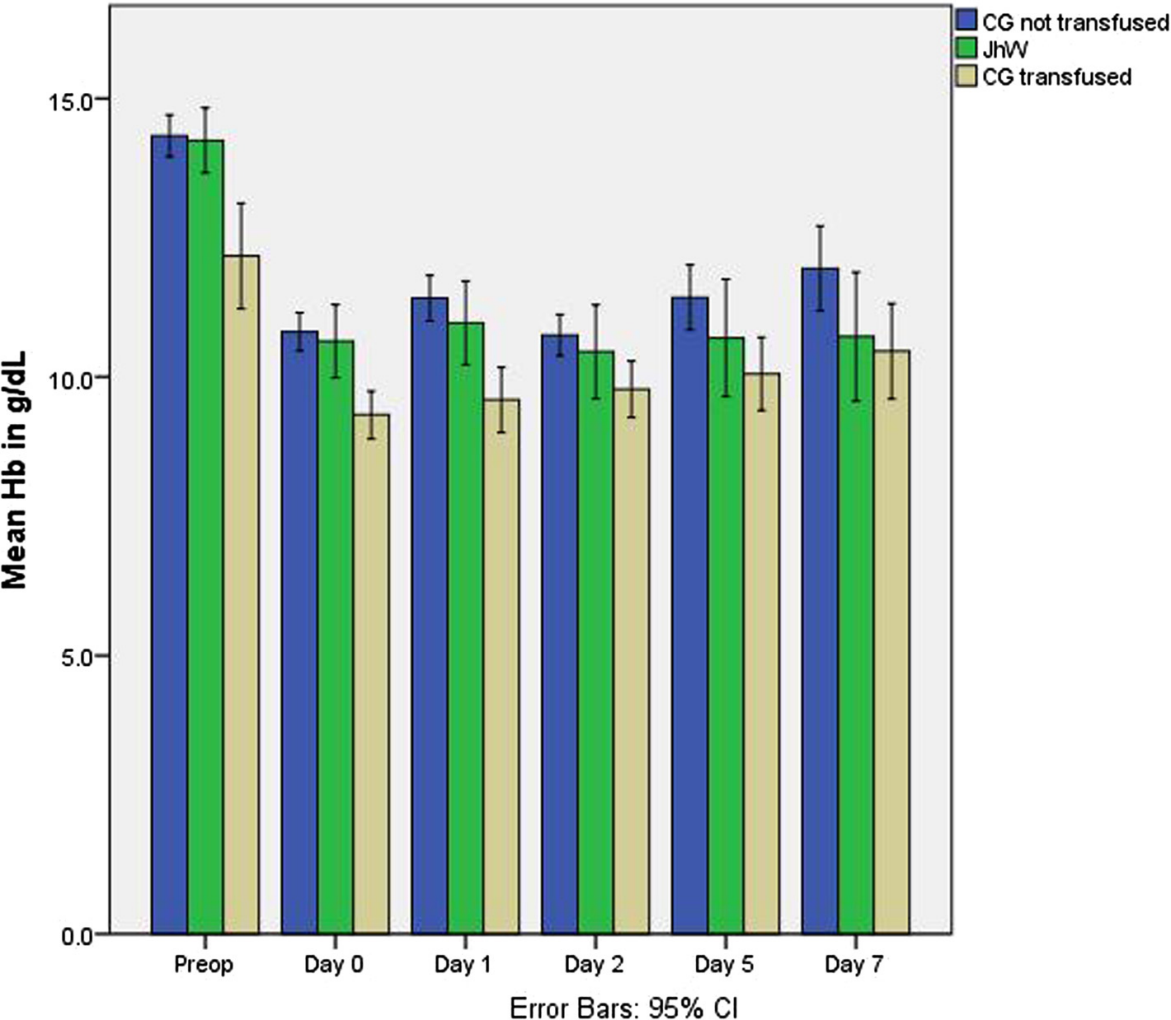
Comparison of mean hemoglobin over time for Control Group transfused, Control Group not transfused, and Jehovah’s Witness patients. (CG = Control Group, JhW = Jehovah’s Witnesses, Hb = Hemoglobin, Preop = Preoperative, CI = Confidence Intervals)

**Fig. 2:**
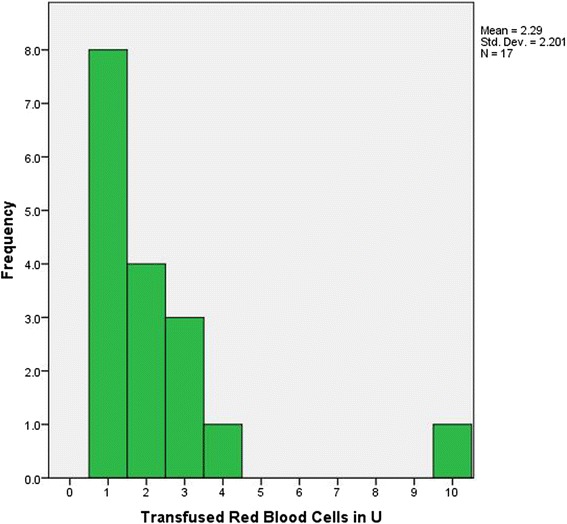
Histogram of transfused Red Blood Cells in Units

There was no significant difference in surgical outcomes. Hospital mortality was 3 % for Jehovah’s Witnesses versus 2 % for control group, *p* = 0.548. The postoperative left ventricular ejection fraction was similar between groups (*p* = 0.912). No stroke was observed in our series. One control patient had acute myocardial infarction but did not require a prolonged hospital stay. One Jehovah’s Witness, who underwent surgery for an off pump coronary artery bypass, required 12 h of intra-aortic balloon pump support immediately after surgery. No significant differences were found in the lengths of the mechanical ventilation support (*p* = 0.218), the intensive care unit stay (*p* = 0.080), or the postoperative stay (*p* = 0.223, Table 4). Only 17 of the 62 (27.4 %) control group patients were transfused with a total of 39 units of red blood cells concentrates (0.63 ± 1.5 units per patient, 2.3 ± 2.2 units per transfused patient). No Jehovah’s Witness patient was transfused (Table 4, Figs. 1 and 2).

## Discussion

In the current study we presented the outcomes after cardiac surgery for a non-selected group of Jehovah’s Witnesses (*n* = 31) operated in our cardiac surgery department from 1991 to 2012. Outcomes and demographic variables were compared with a matched control group (*n* = 62) as described above. No difference was demonstrated in terms of hospital mortality, in-hospital morbidity, or the intensive care or hospital length of stay. Hemoglobin levels remained similar between groups, both pre- and postoperatively.

Previous, non-comparative studies provided evidence that cardiac surgery can be safely performed in Jehovah’s Witnesses, with acceptable outcomes in terms of mortality and morbidity [14–18]. Moreover, a literature search identified four retrospective comparative studies evaluating cardiac surgery in Jehovah’s Witnesses in comparison to a control group of patients. Three studies compared outcomes after cardiac surgery between Jehovah’s Witnesses and a control group of matched transfused patients [4, 6, 7] and another one between Jehovah’s Witnesses and a matched group regardless of blood transfusion [5]. A comparison of the major demographic data and surgical outcomes between our study and previous comparative studies is shown in Table 5.

**Table 5:** Comparison of current study with previous retrospective comparative studies

	Marinakis (*n* = 31 JhWs vs. 62 CG patients)	El Azab [5] (*n* = 123 JhWs vs. 4219 CG patients)	Stamou [7] (*n* = 49 JhWs vs. 196 transfused patients)	Bhaskar [6] (*n* = 49 JhWs vs. 196 transfused patients)	Pattakos [4] (*n* = 322 JhWs vs. 322 transfused patients)
Isolated CABG	15 (48 %)	112 (91 %)	38 (78 %)	25 (51 %)	209 (65 %)
Combined surgery	7 (22.6 %)	10 (8.1)	2 (4 %)	11 (22.4 %)	16 (5 %)
Reoperation	3 (9.7 %)	-	0	-	-
ICU LOS	4.3 ± 3.9 (*p* = 0.08)	2.3 ± 3.2 (*p* = 0.16)	1.5 ± 1.3 (*p* = 0.22)	1 ± 0.4 (*p* = 0.81)	1 (50 % perc) (*p* = 0.001)
In-hospital mortality	1 (3 %) (*p* = 0.55)	3 (2.7 %) (*p* = 0.59)	3 (6 %) (*p* = 0.63)	1 (2 %) (*p* = 0.52)	10 (3 %) (*p* = 0.40)
Postoperative infarct	0 (*p* = 1.00)	2 (1.8 %) (*p* = 0.02)	0 (*p* = 0.62)	0 (*p* = 0.62)	1 (0.3) (*p* = 0.01)

*JhWs* Jehovah’s witnesses, *CABG* coronary artery bypass graft, *ICU* intensive care unit, *LOS* length of stay, *perc* percentiles

Our Jehovah’s Witness group differed significantly from other studies regarding the type of cardiac surgery procedures. Patients underwent a variety of high risk surgical procedures, including urgent operations, reoperations, double valve surgery, combined valve and coronary surgery and ascending aorta procedures (Tables 2, 5). More precisely, in our group only 54 % of all procedures were isolated coronary bypass surgery; whereas, El Azab et al. [5] reported more than 90 % coronary by-pass surgeries for the Jehovah’s Witness group. In the other three studies, which compared Jehovah’s Witnesses to a matched transfused group, coronary bypass surgery accounted for 51 %, 65 %, and 78 % of all cases [4, 6, 7]. Moreover, in our study, 10 % of all procedures were reoperations, which were not reported in the other studies (Table 5).

Despite a trend towards more complex operations, our results are in agreement with previous studies [4–7]. In terms of surgical outcomes, no difference was observed in early operative mortality. There was a trend towards a prolonged intensive care unit length of stay for Jehovah’s Witness group, who stayed 1.3 days more than control group (*p* = 0.08). This difference was in part due to two Jehovah’s Witnesses, who required prolonged intensive care unit hospitalization (14 and 21 days) due to hemoglobin values less than 5.5 g/dL during the postoperative period. Blood restriction was respected according to the patient’s convictions. If these patients were excluded from the analysis as outliners, the new intensive care unit length of stay would be 3.4 ± 1.6 days (*p* = 0.215), which is closer to control group. Nevertheless, we believe that both values should be discussed, as the prolonged intensive care unit stay was related to their refusal of transfusion. Both were released from the hospital uneventfully on postoperative day 28. Both groups of patients had equivalent postoperative lengths of stay. No difference was found with regard to perioperative and postoperative blood loss, suggesting no preferential surgical hemostasis between groups. Two patients had a revision for bleeding in the Jehovah’s Witness group. The first had a preoperative hemoglobin level of 14.4gr/dL. He was reoperated at J0 after a major operation. No apparent source of hemorrhage was found. This patient presented after the reoperation an hemoglobin level of 9.6gr/dL. The patient was discharged uneventfully from the hospital at day 11. The second patient belongs to the group of the two patients exhibiting a hemoglobin level below 5.5gr/dL. He underwent a CABG (coronary arterial bypass graft) procedure and was been reoperated during the following night (12 h after operation). The source of bleeding was in the field of the dissection of the mammary artery. He had a preoperative hemoglobin of 17.3gr/dL. The hemoglobin level after the reoperation was 8gr/dL and at reached its minimal levels of 5.2gr/dL at postoperative day 7. The patient was discharged at day 28. In both groups, drains were withdrawn within the first 48 h. No difference was revealed in terms of postoperative morbidity. The patient who died in the Jehovah’s Witness group was a high risk patient. Preoperative chronic renal insufficiency was the cause of the acute renal failure observed at postoperative day one. Effectively, the mild hemodilution observed during cardiopulmonary bypass doesn’t explain this acute renal failure. The progressive rise in hemoglobin the week following the operation and the occurrence of a general sepsis after a mediastinitis at postoperative day 7 probably exclude that this patient died because of transfusion refusal.

No statistically significant difference was found between groups in the preoperative or postoperative period for hemoglobin and creatinine concentrations or platelet counts (Table 3). Among the control group, the mean preoperative hemoglobin value was significantly less in transfused patients (12.5 ± 2.1 g/dL versus 14.2 ± 1.3 g/dL for the non-transfused control group, *p* = 0.001, Fig. 1). This finding is indicative of the importance of “normal” preoperative hemoglobin levels to reduce the need for red blood cell transfusions [19]. Erythropoietin was administered to 4 Jehovah’s Witnesses preoperatively for low hemoglobin and to 3 more during hospitalization for the same reason. Our center is especially interested in patient blood management strategies; therefore, we adopted a restricted transfusion policy for all the population we are taking in charge. In this study, only 17 of 62 (27.4 %) patients in the control group were transfused with a total of 39 units of red blood cells. Eight of them received only 1 unit of red blood cells and four of them 2 units (Fig. 2). We can reasonably assume that some patients who received only one unit, were transfused to correct a possible hemodilution status frequently present at least until the 3rd postoperative day. These patients could have avoided blood transfusion if a more clinically-oriented approach had been used. Thus, although our transfusion rate is very competitive with regards to the literature, further improvement in blood saving is still possible.

Current literature reports transfusion rates from 25 % to 95 % [1, 2, 11], with an average rate around 50 % [20]. Blood transfusion in cardiac surgery is associated with several adverse effects. A recent meta-analysis concerning the risk of red blood cell transfusions in cardiac surgery reported that transfused patients are at greater risk of developing postoperative infectious complications and acute myocardial infarction, have prolonged length of stay postoperatively in the intensive care unit and hospital, and have increased long term mortality [1]. Moreover, red blood cell transfusion is associated with a significantly increased hospital cost [1]. On the other hand, there are situations where a blood transfusion is lifesaving. It has been reported that hemoglobin levels <7 g/dL in surgical patients who refused blood transfusion, regardless of type of surgery, is associated with higher morbidity and mortality [12]. The same study reported a mortality of more than 40 % for patients with hemoglobin levels <5 g/dL [12]. In our center, we try to respect a threshold of 7 g/dL of hemoglobin for blood transfusions. We accept a relative hemodilution state in the early postoperative period when most blood loss occurs, and we interrupt anti-aggregation therapy 7 days before surgery for planned operations. In addition, we pay major attention in controlling hypertension, especially in the early postoperative period. With this policy, we are able to limit blood transfusion rate after cardiac surgery to 25-30 %.

This study has two relative limitations. The first is that our Jehovah’s Witness group is relatively small and heterogeneous (*n* = 31). On the other hand, it is an unselected group as no Jehovah’s Witness was refused from surgery in our department. In addition, the diversity of the high risk surgical procedures, as stated above, differentiates our group from those evaluated in previous studies. The second limitation is the relatively long period that was considered (from 1991 to 2012). We believe that the algorithm used to select the control group (previous and next patient, of the same sex, undergoing the same procedure) neutralized most of the time effect in the group comparisons. Our results may not be up-to-date with regard to absolute numbers, mainly concerning the length of stay in the hospital and intensive care unit, as there is a tendency over time for faster hospital discharges, but the between groups comparisons are probably not affected due to design of our study. Furthermore, as our hospital has no intermediate unit between the intensive care unit and the normal ward, our intensive care unit length of stay is relatively prolonged in comparison to other studies. On the other hand, our group of Jehovah’s Witnesses was a non-selected group, characterized by more complex cardiac procedures, than reported in current literature, with 10 % of reoperations.

## Conclusions

The results of our study support the view that cardiac surgery, including complex procedures and reoperations, can be performed in Jehovah’s Witnesses provided rigorous preoperative preparations, perioperative hemostasis, and postoperative management. Moreover, we confirmed that there is no difference in terms of early postoperative mortality or serious morbidity between Jehovah’s Witnesses and non-Jehovah’s Witness populations. It would be of interest to compare the long term mortality between Jehovah’s Witnesses and non-Jehovah’s Witnesses after cardiac surgery in future studies.

### Ethics approval and consent to participate

This study was approved by the Brugmann University Hospital Institutional Review Board. Individual consent was waived by the committee because of the retrospective nature of the study.

## Abbreviations

CABG: coronary arterial bypass graft
FiO2: inspired fraction in oxygen
SvO2: venous saturation in oxygen
